# Phase-separated super-enhancers confer an innate radioresistance on genomic DNA

**DOI:** 10.1093/jrr/rrae044

**Published:** 2024-06-14

**Authors:** Koki Matsumoto, Dini Kurnia Ikliptikawati, Kei Makiyama, Kako Mochizuki, Maho Tobita, Isao Kobayashi, Dominic Chih-Cheng Voon, Keesiang Lim, Kazuma Ogawa, Ikuo Kashiwakura, Hiroshi I Suzuki, Hironori Yoshino, Richard W Wong, Masaharu Hazawa

**Affiliations:** Division of Transdisciplinary Sciences, Graduate School of Frontier Science Initiative, Kanazawa University, Kakuma-machi, Kanazawa, Ishikawa 920-1192, Japan; WPI Nano Life Science Institute, Kanazawa University, Kanazawa, Ishikawa 920-1192, Japan; Division of Transdisciplinary Sciences, Graduate School of Frontier Science Initiative, Kanazawa University, Kakuma-machi, Kanazawa, Ishikawa 920-1192, Japan; Faculty of Biological Science and Technology, Institute of Science and Engineering, Kanazawa University, Kakuma-machi, Kanazawa, Ishikawa 920-1192, Japan; Faculty of Biological Science and Technology, Institute of Science and Engineering, Kanazawa University, Kakuma-machi, Kanazawa, Ishikawa 920-1192, Japan; Faculty of Biological Science and Technology, Institute of Science and Engineering, Kanazawa University, Kakuma-machi, Kanazawa, Ishikawa 920-1192, Japan; Division of Transdisciplinary Sciences, Graduate School of Frontier Science Initiative, Kanazawa University, Kakuma-machi, Kanazawa, Ishikawa 920-1192, Japan; Institute for Frontier Science Initiative, Kanazawa University, Kakuma-machi, Kanazawa, Ishikawa 920-1192, Japan; Cancer Research Institute, Kanazawa University, Kakuma-machi, Kanazawa, Ishikawa 920-1192, Japan; WPI Nano Life Science Institute, Kanazawa University, Kanazawa, Ishikawa 920-1192, Japan; Division of Transdisciplinary Sciences, Graduate School of Frontier Science Initiative, Kanazawa University, Kakuma-machi, Kanazawa, Ishikawa 920-1192, Japan; Institute for Frontier Science Initiative, Kanazawa University, Kakuma-machi, Kanazawa, Ishikawa 920-1192, Japan; Faculty of Pharmaceutical Sciences, Institute of Medical, Pharmaceutical, and Health Sciences, Kanazawa University, Kakuma-machi, Kanazawa, Ishikawa 920-1192, Japan; Department of Radiation Science, Hirosaki University Graduate School of Health Sciences, 66-1 Hon-cho, Hirosaki, Aomori 036-8564, Japan; Division of Molecular Oncology, Center for Neurological Diseases and Cancer, Nagoya University Graduate School of Medicine, 65 Tsurumai-cho, Showa-ku, Nagoya, Aichi 466-8550, Japan; Institute for Glyco-Core Research (iGCORE), Nagoya University, 65 Tsurumai-cho, Showa-ku, Nagoya, Aichi 464-8601, Japan; Center for One Medicine Innovative Translational Research (COMIT), Nagoya University, 65 Tsurumai-cho, Showa-ku, Nagoya, Aichi 464-8601, Japan; Department of Radiation Science, Hirosaki University Graduate School of Health Sciences, 66-1 Hon-cho, Hirosaki, Aomori 036-8564, Japan; Division of Transdisciplinary Sciences, Graduate School of Frontier Science Initiative, Kanazawa University, Kakuma-machi, Kanazawa, Ishikawa 920-1192, Japan; WPI Nano Life Science Institute, Kanazawa University, Kanazawa, Ishikawa 920-1192, Japan; Faculty of Biological Science and Technology, Institute of Science and Engineering, Kanazawa University, Kakuma-machi, Kanazawa, Ishikawa 920-1192, Japan; Institute for Frontier Science Initiative, Kanazawa University, Kakuma-machi, Kanazawa, Ishikawa 920-1192, Japan; Division of Transdisciplinary Sciences, Graduate School of Frontier Science Initiative, Kanazawa University, Kakuma-machi, Kanazawa, Ishikawa 920-1192, Japan; WPI Nano Life Science Institute, Kanazawa University, Kanazawa, Ishikawa 920-1192, Japan; Faculty of Biological Science and Technology, Institute of Science and Engineering, Kanazawa University, Kakuma-machi, Kanazawa, Ishikawa 920-1192, Japan; Institute for Frontier Science Initiative, Kanazawa University, Kakuma-machi, Kanazawa, Ishikawa 920-1192, Japan

**Keywords:** phase separation, super-enhancer, radiation damage, brd4

## Abstract

Recently, biomolecular condensates formed through liquid–liquid phase separation have been widely reported to regulate key intracellular processes involved in cell biology and pathogenesis. BRD4 is a nuclear protein instrumental to the establishment of phase-separated super-enhancers (SEs) to direct the transcription of important genes. We previously observed that protein droplets of BRD4 became hydrophobic as their size increase, implying an ability of SEs to limit the ionization of water molecules by irradiation. Here, we aim to establish if SEs confer radiation resistance in cancer cells. We established an *in vitro* DNA damage assay that measures the effect of radicals provoked by the Fenton reaction on DNA integrity. This revealed that DNA damage was markedly reduced when BRD4 underwent phase separation with DNA. Accordingly, co-focal imaging analyses revealed that SE foci and DNA damage foci are mutually exclusive in irradiated cells. Lastly, we observed that the radioresistance of cancer cells was significantly reduced when irradiation was combined with ARV-771, a BRD4 de-stabilizer. Our data revealed the existence of innately radioresistant genomic regions driven by phase separation in cancer cells. The disruption of these phase-separated components enfolding genomic DNA may represent a novel strategy to augment the effects of radiotherapy.

## INTRODUCTION

A range of biomolecules have the propensity to form condensates via a phenomenon known as liquid–liquid phase separation (LLPS), where a homogeneous solution of macromolecules separates into a dense phase and a dilute phase without a membrane boundary [1]. LLPS plays a crucial role in the spatial and temporal organization of biomolecules for proper functions within cells [2–4].

Inside the nucleus, LLPS drives the formation of large transcription apparatuses called super-enhancers (SEs)—clusters of enhancers occupied by extraordinarily high densities of transcription factors (TFs) and cofactors (e.g. the Bromodomain and Extraterminal (BET) proteins, mediators)— to regulate important lineage-determining genes [5–7]. Mechanistically, BRD4, a member of the BET protein family, binds acetylated lysine residues on histone (H3K27ac) via its bromodomains located at its amino (N)-terminus. At the same time, the phase separation of transcription machinery is driven in part by the intrinsically disordered regions (IDRs) of BRD4, mediators and TFs [5, 6]. Of note, *BRD4* encodes two main isoforms, BRD4 long (BRD4-L) and BRD4 short (BRD4-S), and only the BRD4-L harbors the SE determinant IDRs [6].

So far, divergent biological roles of BRD4 isoforms in transcription regulation, DNA damage response and cancer progression have been reported [8–11]. BRD4 isoforms have distinct functions in the DNA damage response. The BRD4-L promotes the non-homologous end joining DNA repair pathway [12], while the BRD4-S, with unique short 75 amino-acid segments, inhibits DNA damage signaling by chromatin compaction [10]. Recently, we demonstrated that BRD4-IDRs condensates become hydrophobic and viscous as they increase in size by *in vitro* experiment [13]. This observation raises the possibility that SEs have physicochemical roles in limiting the hydrolysis of water upon radiation exposure. In addition, we wondered whether the phase-separated dense droplets even insulate chromatin from reactive oxygen species (ROS).

Radiation therapy is a powerful treatment modality for eradicating cancer cells through the induction of DNA damage using ionizing radiation, such as high-energy photon radiation, X-rays and gamma rays. In addition to the direct action of ionizing radiation on DNA, ROS, derived as the radiolysis product of water, largely attack DNA [14, 15]. To date, the alterations of cellular functions (i.e. DNA repair activity, redox activity, pro-survival signaling pathway) and tumor microenvironment have been attributed to radioresistance [15–19]. However, the effects of LLPS-mediated hydrophobicity on DNA damage remain undetermined. Hence, we sought to dissect the physicochemical roles of phase-separated SEs during ROS- or IR-induced DNA damage. Unless otherwise stated, the BRD4 mentioned in this study refers to BRD4-L only.

## MATERIALS AND METHODS

### Cell culture

The HCT116, A549 and TE5 cancer cell lines were maintained in DMEM (HCT116 and A549) or RPMI1640 (TE5) medium supplemented with 10% (vol/vol) fetal bovine serum and 1% (vol/vol) penicillin/streptomycin. All cells were cultured at 37°C, 5% CO_2_ in a humidified atmosphere.

### Drug treatment

Cells (1.0 × 10^5^) were seeded on 35-mm culture dishes and cultured for 2 days. For treatment, cells were replenished with fresh medium with 1 μM ARV-771 (Selleck, # S8532) or dimethyl sulfoxide (DMSO) and further cultured for 24 h. During harvest, cells were washed with phosphate-buffered saline (PBS) and used in subsequent experiments.

### siRNA transfection

Knockdown of BRD4 in HCT-116 cells was achieved using siRNA targeting BRD4 (sc-43 639, Santa Cruz Biotechnology, Inc.) and RNAiMAX (Thermo Fisher Scientific, Inc.) according to the manufacturer’s protocol. Silencer^®^ Select Negative Control #1 (4390843, Thermo Fisher Scientific, Inc.) was used as the negative control. The final concentration of siRNA was 100 nM. After 48 h of transfection, cells were harvested.

### X-ray irradiation

For the microscopic imaging and surviving fraction experiments, the cells were irradiated with X-ray (150 kVp, 20 mA, 0.5-mm Al and 0.3-mm Cu filters) using an X-ray generator (MBR-1520R-3; Hitachi Medical Corporation). The dose rate was 0.99–1.01 Gy/min. For the single cell gel electrophoresis, tripsinized cells were placed in a 35 mm Petri dish in ice-cold PBS, and cells were irradiated with X-ray on a Faxitron RX-650 (Faxitron, 130 kVp, 1.04 Gy/min) for 115 s (2 Gy).

### Clonogenic survival assay

Cells treated with ARV-771 or transfected BRD4-specific siRNA (sc-43 639, Santa Cruz) were irradiated with X-ray and cultured for 24 h. Cells were then counted by trypan blue dye exclusion assay, seeded on 60-mm culture dish and cultured for 8–12 days. Subsequently, the cells were fixed with methanol and stained with Giemsa solution (Wako), and colonies with >50 cells were counted. Experiments were performed in triplicate and repeated three times independently (*n* = 3). Data are presented as mean ± SE. Statistical analyses (Student’s *t*-test or Welch’s *t*-test based on the data distribution) were performed using GraphPad Prism 7 software. *P* < 0.05 was considered statistically significant.

### Protein purification

The BRD4-IDR-encoding pET plasmid was transformed into the ArcticExpress (Agilent) *Escherichia coli* strain and cultured as recently reported [20]. Briefly, a single bacterial colony was cultured in Luria-Broth (LB) media containing kanamycin for 16 h at 37°C. The starter culture was then diluted 1:50 in 1 L LB solution and cultured for 3 h at 30°C. Isopropyl β-D-thiogalactopyranoside (IPTG) was then added to 1 mM and the culture was maintained for 24 hat 13°C. The harvested bacteria were resuspended in 30 ml buffer A (50 mM Tris HCl pH 7.5, 500 mM NaCl, 10 mM Imidazole) with protease inhibitors and lysed with a French press. After centrifugation, the lysate supernatant was applied to a pre-equilibrated His Trap HP 1 ml (Cytiva). The column was washed once with buffer A and eluted with buffer B (50 mM Tris HCl pH 7.5, 500 mM NaCl, 200 mM Imidazole) with protease inhibitors. Eluted proteins were concentrated using a centrifugal filter (Amicon Ultra). Glycerol was added to the purified protein to 10% and stored at −80°C.

### *In vitro* DNA damage assay

The pEGFP-N1 (Clontech Cat#V012021) plasmid was digested with NheI (Clontech #1241A) to release a 4733 bp fragment. This was diluted in 20 mMTris-HCl pH 7.5 to 5 ng/μl and incubated with 2.5 μM recombinant BRD4-IDR–mEGFP fusion protein or 10% PEG8000 (Sigma) in the presence or absence of 10 mM hydrogen peroxide (Nacalai Tesque, #18411) to form droplets. The mixture was immediately incubated at 37°C for 30 min after the addition of 5 μM ferrous sulfate heptahydrate (Nacalai Tesque #19532), with or without 10 mM hydrogen peroxide. To digest the proteins, the mixture was diluted in water, and Proteinase K (Cell Signaling Technology (CST), #10012) was added to a final concentration of 250 μg/ml, along with 187.5 mM NaCl (CST, #7010), and incubated at 65°C for 2 h. The DNA was then purified (FastGene, #FG-91302), quantified on a NanoDrop LITE Spectrophotometer (Thermo) and subjected to 1% agarose gel electrophoresis to assess the extent of DNA damage.

### *In vitro* droplet assay

The recombinant protein BRD4-IDR and linearized pEGFP-N1 were diluted to a final concentration of 5 μM and 5 ng/μl, respectively, in droplet buffer (50 mM Tris–HCl pH 7.5, 10% glycerol, 1 mM DTT) containing the desired NaCl concentration, with or without 10% PEG, then immediately loaded on a glass-bottom dish (MATSUNAMI) and covered with a coverslip. Images were captured on a Leica TCS SP8 confocal microscope (×63/1.40 objective) and processed for nMDP analysis [21] using Fiji Image J using default parameters [22].

### SDS-PAGE and western blotting

SDS-PAGE analysis and western blotting were performed as previously reported [23]. The following antibodies were used: anti-BRD4 antibody (CST, #13440), anti-actin antibody (CST, #4967) and horseradish peroxidase (HRP)-linked anti-rabbit IgG antibody (CST, #7074). Antigens were visualized using Clarity™ Western ECL Substrate (Bio-Rad).

### cDNA preparation and quantitative real-time RT-PCR assay

We used 500 ng RNA for cDNA preparation using ReverTra Ace^®^ qPCR RT Master Mix (TOYOBO). Quantitative real time RT-PCR was performed by KOD SYBR qPCR™ Mix (TOYOBO) in an Applied Biosystems™ StepOne™ Real-Time PCR System (Thermo Fisher Scientific) according to the manufacturer’s instructions. The relative mRNA expression level of target genes was calculated using GAPDH as a loading control. The amplification of target regions was performed with primers: GAPDH-F:5′-GAAGGTGAAGGTCGGAGTC-3′; GAPDH-R:5′-GAAGATGGTGATGGGATTTC-3′;matured-MYC-F:5′-ACTTCTACCAGCAGCAGCAG-3′; matured-MYC-R:5′-GAGCAGAGAATCCGAGGACG-3′; nascent MYC-F:5′-AAAAGGGAGGCGAGGATGTG-3′; nascent MYC-R:5′-GGGCTTTAACACCCCTCCAT-3′.

### Immunofluorescence analysis

Cells for immunofluorescence (IF) analysis were treated as previously reported [24]. Briefly, cells on coverslips were fixed for 10 min in 4% paraformaldehyde diluted in PBS for 10 min, then permeabilized with 0.3% Triton X-100 in PBS for 3 min at RT and blocked with PBS containing 4% bovine serum albumin (BSA) and 0.1% Tween-20. The fixed cells were incubated with antibodies against histone H2A.X (γH2AX; phosphor-Ser139; CST, #9718), Acetyl-Histone H3K27 (Lys27; CST, #8173) or BRD4 (CST, #63759)) for 2 h. Cells were then washed three times and incubated with Alexa Fluor-conjugated secondary antibody (Life Technologies) for 1 h and mounted with Pro-Long Gold Antifade reagent (Life Technologies) and DRAQ5™ (Biostatus). Images were captured on a Leica TCS SP8 confocal microscope (×100/1.4 objective) and processed for nMDP analysis [21] or quantification of the number of γH2AX foci using Fiji Image J using default parameters [22].

### Chromatin immunoprecipitation sequencing data analysis

Chromatin immunoprecipitation sequencing (ChIP-Seq) data for γH2AX derived from irradiated HeLa cells were obtained from GEO Accession GSE60526 [non-irradiated cells (GSM1481674) and 0.5 h irradiation (GSM1481676)]. Moreover, HeLa ChIP-Seq datasets from GSE151038 containing GSM4564684 (BRD4) and GSM4564682 (Input) and GSE51334 containing GSM733684 (H3K27ac) and GSM822286 (MYC) were also extracted. GSM1481688 was applied as input to determine significant peaks of H3K27ac [25–27]. ChIP-Seq reads were aligned to the hg19 genome assembly using Bowtie2 with the default parameters. Only tags that uniquely mapped to the genome were used for further analysis. PCR duplicates were removed using the Picard tools. Peaks were identified with the homer findPeaks.pl script. Peak annotation was processed with the homer annotatePeaks.pl script. Heatmaps were plotted using deeptools. Bigwig files were generated using the deepTools bamCoverage function with—normalizeUsing RPGC—effectiveGenomeSize 2 864 785 220—binSize 1. Peak annotation and bed files were obtained using HOMER. Heatmap analysis was performed using deeptools.

### Neutral comet assay

Assessment of DNA damage and DNA double-strand break (DSB) formation in cells was performed using the CometAssay single cell gel electrophoresis assay (Trevigen). Cells were harvested and resuspended in low-melting point agarose, plated onto provided glass slides and subjected to electrophoresis in neutral electrophoresis buffer (100 mM Tris, 300 mM Na Acetate, pH 9.0). Slides were processed according to the manufacturer’s instructions. DNA tails were visualized after SYBR Gold staining using a Zeiss LSM5 EXCITER microscope with a mercury lamp and quantified using ImageJ software with the OpenComet [28].

## RESULTS

### Establishment of an *in vitro* DNA damage assay

**Fig. 1:**
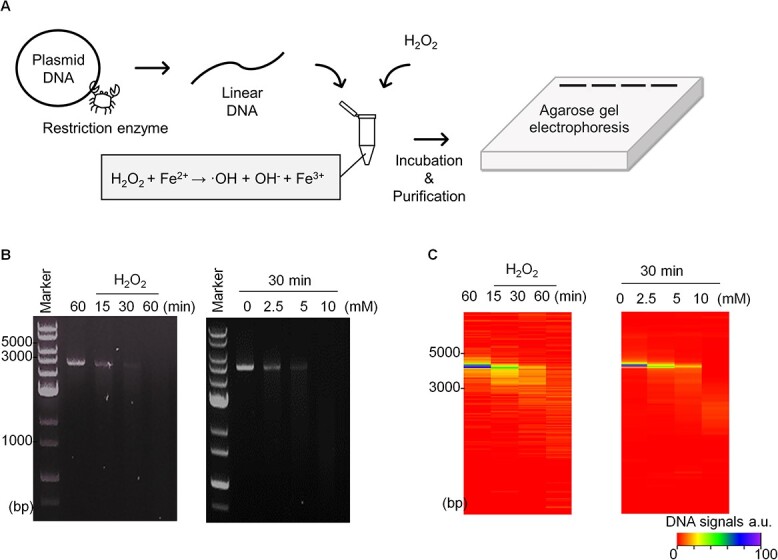
Establishment of *in vitro* DNA damage assay. (**A**) Scheme illustrating the preparation of linear DNA, ROS generation and quantification of DNA damage using electrophoresis. (**B**) Result of DNA damage represented in reaction time-dependent manner (left) and concentration-dependent manner (right). (**C**) Heatmap visualization of the extent of DNA amounts. Maximum signal of DNA (non-damaged DNA) was prepared as 100%.

To biochemically replicate the effects of ionizing radiation, an *in vitro* DNA damage assay was established, where hydroxyl radicals generated via the Fenton reaction cleaved a linear double-stranded DNA fragment (Fig. 1A). Subsequently, we quantified the cleaved DNA by agarose gel electrophoresis. As expected, the extent of damage was proportional to either the reaction time (Fig. 1B and C, left) or H_2_O_2_ concentration (Fig. 1B and C, right), which provided a means to quantify the effects of phase-separated proteins.

### Phase-separated BRD4-IDRs shielded DNAs from radicals

**Fig. 2:**
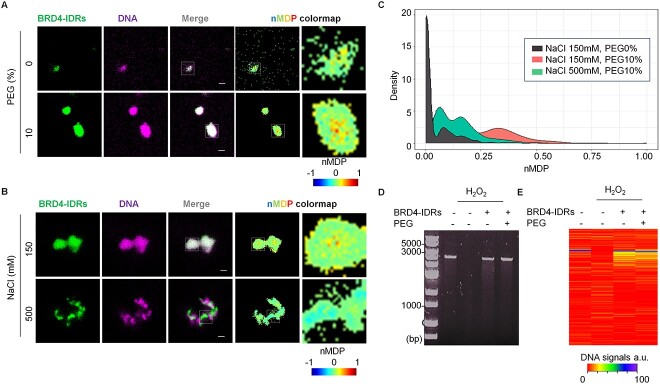
Phase-separated BRD4-IDRs minimize the DNA damage *in vitro*. (**A**) BRD4-IDRs were diluted in the buffer to a final concentration of 5 μM in the presence of DNA (5 ng/μl) and indicated conditions. Colocalization in ROI was visualized using nMDP colormap. (**B**) BRD4-IDRs and DNA were diluted in the buffer to a final concentration of 5 μM and indicated conditions in the presence of 10% PEG. (**C**) The extent of colocalization was quantified using nMDP value and visualized by density plot. (**D**) Result of DNA damage represented in the presence of BRD4-IDRs with/without 10% PEG. (**E**) Heatmap visualization of the extent of DNA amounts. Maximum signal of DNA (non-damaged DNA) was prepared as 100%.

To understand the biological roles of phase separation in DNA damage responses, we applied BRD4-IDRs in *in vitro* DNA damage assays, in the presence or absence of polyethylene glycol (PEG), a crowding agent known to promote the separation of protein phases [29]. We first analyzed the interaction between BRD4-IDRs and DNAs using fluorescent microscopy, where we observed their aggregation upon mixing (Fig. 2A). In the presence of PEG, DNAs were effectively compartmentalized into phase-separated BRD4-IDRs droplets (Fig. 2A). This was likely achieved via electrostatic interactions as high salt conditions blocked the interaction between BRD4-IDRs and DNAs (Fig. 2B). The extent of their colocalizations was determined by quantifying the normalized mean deviation product (nMDP) of the captured images (Fig. 2A–C). This method quantitatively creates a spatial map of colocalization of two fluorescent signals of interest. The nMDP values range from −1.0 to 1.0, wherein values above 0 indicate the fraction of positively correlated (colocalized) pixels (in detail, see Gorlewicz *et al.* (21)). Through this, it was revealed that BDR4-IDRs and DNAs readily undergo phase separation together in the presence of polyethylene glycol (PEG). We next applied the assay to compare the levels of DNA damage under these conditions and observed that the presence of BRD4-IDRs significantly prevents DNA damage (Lane3 vs Lane4 in Fig. 2D and E). Moreover, the protective effect of BRD4-IDRs was further enhanced under droplet-phase (Fig. 2D and E), implicating a role for phase-separation in protecting DNA from damage.

### BRD4 condensates prevent DNA DSBs upon radiation exposure

**Fig. 3:**
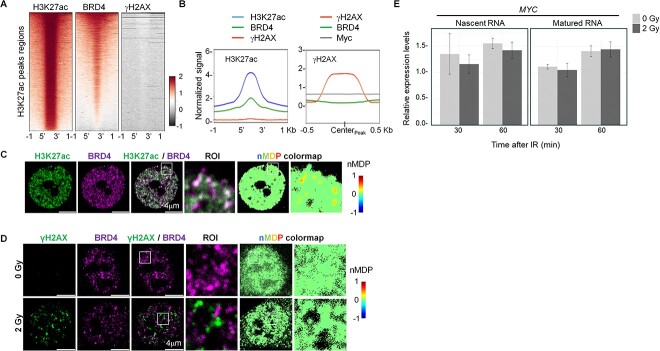
Genomic DNA occupied by BRD4 is resistant to DNA damage upon IR. (**A**) Heatmaps of ChIP-Seq signals at H3K27ac peak regions (±1 kb of peak region). (**B**) Line plots showing the distribution of indicated ChIP-Seq signals at H3K27ac (left; ±1 kb of peak region) and γH2AX (right; ±0.5 kb of peak centers). (**C**, **D**) IF imaging of H3K27ac and BRD4 in TE5 cells (C), γH2AX and BRD4 in HCT116 cells (D). Colocalization in ROI was visualized using nMDP colormap. (**E**) qRT-PCR analysis of the levels of MYC transcripts using cDNA prepared from non-IR and irradiated HCT-116 cells. A non-irradiated sample at 0 min was used as a reference point. The Intronic primer set was used to detect nascent RNA (left), and an exonic primer set was used to detect matured RNA. Data show mean ± SEM from three independent experiments (*n* = 3).

**Fig. 4:**
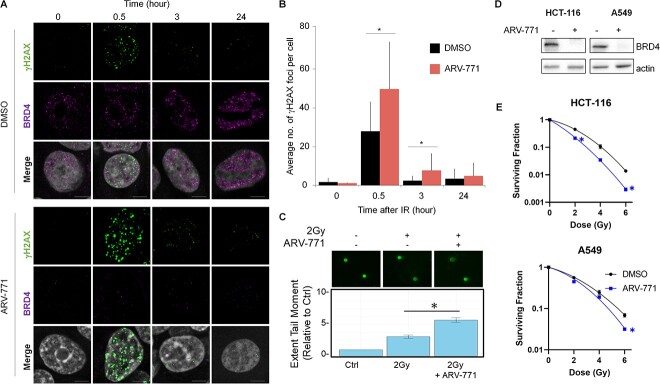
Pharmacological degradation of BRD4 works as radiation sensitizer. (**A**) Representative images of BRD4 and IR-induced γH2AX in the cells with either 2 Gy IR alone or a combination of IR and BRD4 degradation. (**B**) Quantification of γH2AX foci (30 cells) based on a single experiment. Similar results were obtained in two independent experiments. Data show mean ± SD. (**C**) Fluorescence microscopy images of neutral comet assay in cells upon 2 Gy pretreated for 12 h with DMSO or ARV-771 (*n* = 3). The bar graph shows mean ± SEM. Significance was assessed using a Student’s *t*-test (*P < 0.05). (**D**) Western blotting analysis of BRD4 upon ARV-771 treatment in HCT116 and A549 cell lines. (**E**) Survival fraction of HCT116 and A549 cell lines upon IR alone and IR combined with ARV-771.

IDRs of BRD4 play a pivotal role in establishing discrete bodies of SE condensates. Therefore, we asked whether genomic regions organized as SEs would avoid forming DSBs in irradiated cells. To define the sites of DSBs, we surveyed the distribution of γH2AX, an established histone modification that mainly occurs upon DSBs [14, 17]. We interrogated ChIP-Seq dataset generated from irradiated HeLa cells [26] and compared the occupancy of γH2AX as well as BRD4 on the genomic regions marked by histone H3K27ac (Fig. 3A). Importantly, BRD4, but not γH2AX, co-localized with histone H3K27ac at putative enhancer regions (Fig. 3A and B). Reciprocally, BRD4 and MYC, a pioneering TF for SEs formation [30], collectively occupied regions that are void of putative DSBs (Fig. 3B). Consistent with these ChIP-Seq data, we irradiated HCT116 cells and studied the spatial distribution of BRD4 and γH2AX and found that BRD4 colocalized with H3K27ac (Fig. 3C) but was mutually exclusive to γH2AX binding (Fig. 3D). This mutual exclusiveness of BRD4 and γH2AX was further supported by the fluorescent intensity profiles along a line at multiple regions inside the nucleus (Supplementary Fig. 1). Furthermore, to ask whether SE-relevant genomic regions are intact upon radiation exposure, we quantified the amounts of nascent RNA transcript from *MYC*, a bona fide oncogene driven by SE in several types of cancers. This analysis found that the level of nascent transcripts of *MYC* seldom changed after irradiation, suggesting that SE genomic regions are intact (Fig. 4E). Collectively, these results suggested that genomic regions containing SEs are resistant to radiation-induced DSBs.

### The degradation of BRD4 sensitizes cancer cells to radiation

**Fig. 5:**
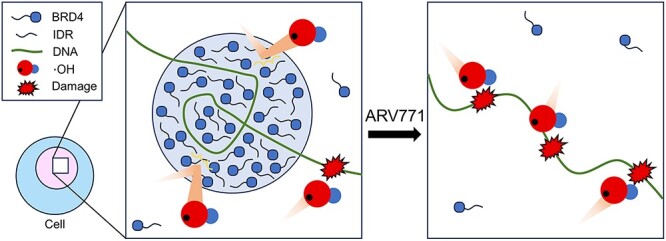
A model whereby phase-separated SEs prevent DNA damage from radiation or ROS.

To ascertain the physiological impact of the abovementioned observations, we investigated whether the disruption of SEs would augment the radiosensitivity of cancer cells. To do this, ARV-771, which is a small chemical that promotes BRD4 degradation [31], was employed to induce the collapse of SEs, and the kinetics of γH2AX of irradiated cells with BRD4 degradation was evaluated by microscopic imaging analysis (Fig. 4A). This approach demonstrated that the combination of IR and ARV-771 significantly increased the number of γH2AX foci compared to IR alone (Fig. 4A and B). To define whether the increase of gH2AX was from a faulty DNA repair pathway or an increase of DSB, we first investigated the expression levels of the gene involved in the DNA repair pathway. Re-analysis of public RNA-seq data [32] demonstrated that ARV-771 barely altered the expression levels of DSB repair genes (Supplementary Fig. 2). In addition, we also performed WB to see Ku80 protein levels in HCT116 and TE5 cells treated with ether ARV-771 or another selective BET bromodomain inhibitor, JQ1 (Supplementary Fig. 2). These BET inhibitors reduced the protein amounts of MYC, a bona fide SE target; however, protein amounts of KU80 seldom changed. These data indicate that BRD4 degradation or functional inhibition of BRD4 rarely affects expression levels of DNA repair-related genes. Next, to evaluate the yields of DSB, we prepared HCT116 cells either with or without BRD4 degradation and proceeded them for neutral comet assay immediately after 2 Gy IR (Fig. 4C). Importantly, the DSB yields significantly increased in the combination of IR and BRD4 degradation comparing to that in IR alone (Fig. 4C). Collectively, these results suggested that BRD4 droplets prevent DSB formation upon radiation exposure (Fig. 4C). Finally, the surviving fraction of irradiated cancer cells combined with BRD4 degradation was evaluated. As shown in Fig. 4D and E, co-treatment with ARV-771 significantly reduced the surviving fraction of irradiated cancer cells (Fig. 4B). Not surprisingly, the depletion of BRD4 by siRNA phenocopied the effect of ARV-771 on the surviving fraction of irradiated cells (Supplementary Fig. 3). Collectively, these data suggested that the abolishment of phase-separated SEs may have therapeutic merits by sensitizing cancer cells to radiation therapy (Fig. 5).

## DISCUSSION

In this study, we established that genomic regions with phase-separated SEs are radioresistant. Importantly, the pharmaceutical degradation of BRD4 abolished SEs and increased DSBs, thereby improving the effects of radiation-induced cell death. Our data highlight a hitherto unappreciated role of phase separation on the DNA damage response upon IR.

Phase separation is involved in the regulation of such diverse biological processes as intracellular signaling, transcription, chromatin organization and DNA damage response [33]. Upon DNA damage, DNA repair-related proteins form liquid-like droplets at damage sites through phase separation to promote DNA repairs [34, 35]. Our study demonstrated that, in addition to DNA repair, phase-separated SEs conferred intrinsic radioresistance to genomic DNA, which may in turn limit radiosensitivity of cancer cells. DNA exhibits diverse structural states within the nucleus. Chromatin, the packaged and organized form of DNA, can transition between open, accessible regions and condensed, nucleosome-bound segments according to histone modification patterns [36]. BRD4 explicitly targets the chromatin marked by H3K27ac and establishes phase-separated SEs through IDRs–IDRs interaction [6, 37]. In addition to SEs, several phase-separated structures are present within the nucleus, including nucleolus, cajal bodies, paraspeckles, etc. [38]. It would be of interest to ascertain the radiosensitivity within these structures in future studies.

The current study demonstrates a physicochemical function of phase-separated BRD4 to limit DSBs upon either oxidative stress or irradiation; and that pharmaceutical degradation of BRD4 significantly reduced the clonogenic growth potential of irradiated cancer cells. Generally, BRD4 controls genes involved in cell cycle progression and lineage allocations by chromatin remodeling and transcriptional regulation, and its aberrant activity is implicated in various types of cancers [30, 39]. A recent study further delineates a role in maintaining genomic integrity, whereby the loss of BRD4 in cancer cells induced DNA damage and cell death due to an accumulation of transcription-replication conflicts and failure of checkpoint signaling [25]. Considering the multiple biological functions of BRD4, how the SEs may render sensitive to IR in relations to those functions deserves further exploration.

As conclusion, we determined a significant role for LLPS to confer resistance to IR or oxidative stresses on DNA. The disruption of phase-separated SEs augmented the therapeutic effects of IR, offering an informative snapshot of phase separation in radiation biology as well as free radical biology.

## CONFLICT OF INTEREST

The authors declare no competing financial interest.

## FUNDING

This work was supported by an Infiniti Grant and in part by Grants-in-Aid for scientific research (JSPS KAKENHI Grant Number 23H04278 to R.W.W. and 23H02853 to M.H.) from the Japan Society for the Promotion of Science. This research was supported by AMED under Grant Number JP23kk0305026 to H.I.S., and the grant provided by The Sumitomo Foundation to M.H., SGH Cancer Research Grant to M.H., Exploratory Research Grant for Young Scientists Hirosaki University to H.Y., The Nakatomi Foundation to M.H. and Life Science Foundation of Japan to M.H.

## Supplementary Material

Supplemental_Imformation_rrae044

## Notes

Presentation at a conference: The 65th Annual Meeting of the Japanese Radiation Research Society.
