# Supplementary material for: Phase-separated super-enhancers confer an innate radioresistance on genomic DNA
Source: J Radiat Res. 2024 Jun 14;65(4):482–90. doi: 10.1093/jrr/rrae044 (PMC11262858; doi:10.1093/jrr/rrae044)
Supplement: Supplemental_Imformation_rrae044 [file supplemental_imformation_rrae044.pdf]

Supplemental Figure S1

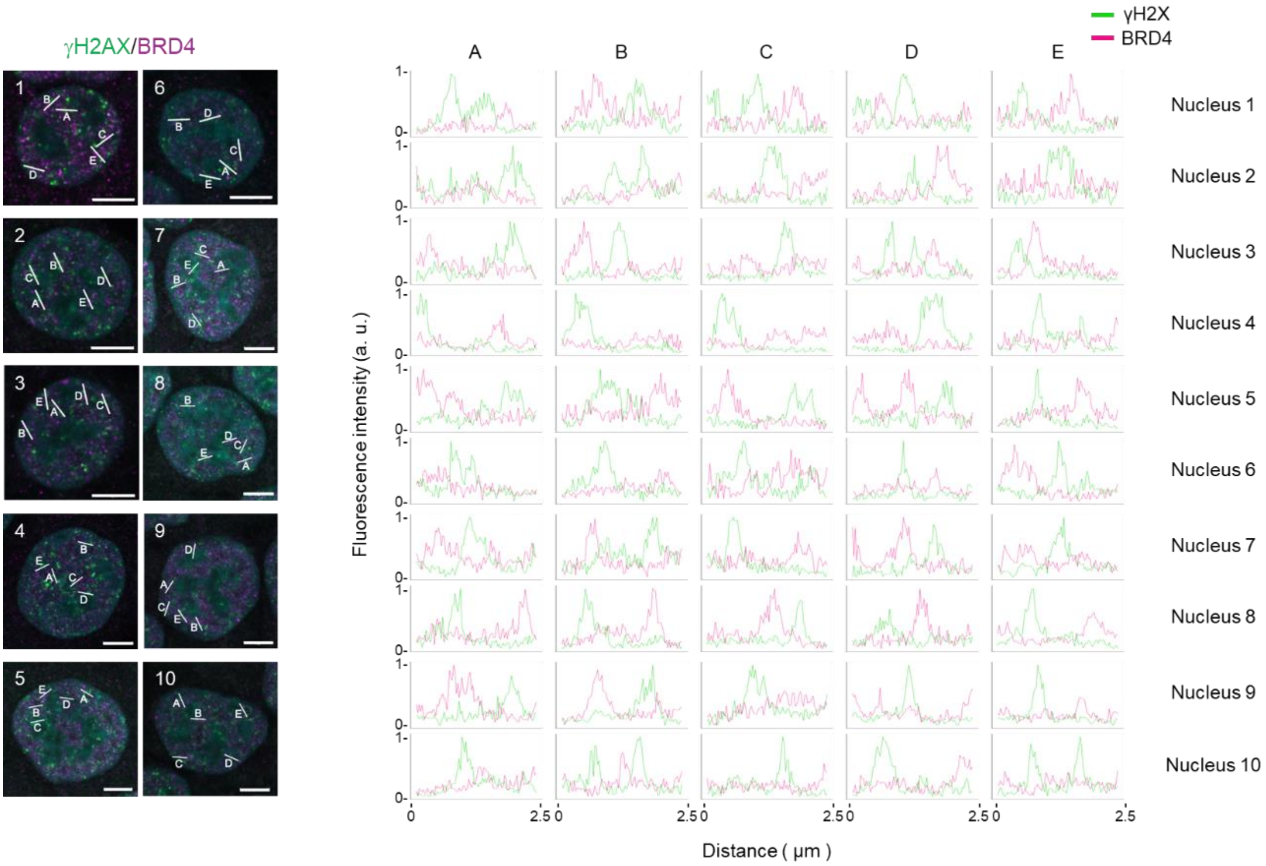

Supplemental Fig. 1. The fluorescence intensity profiles of γH2AX and BRD4

Representative images of γH2AX (green) and BRD4 (purple) at 30 min post 2Gy IR were shown in left. Bar = 5μm. The intensity profiles of each signal along an indicated line were shown in right.

Supplemental Figure S2

(A)

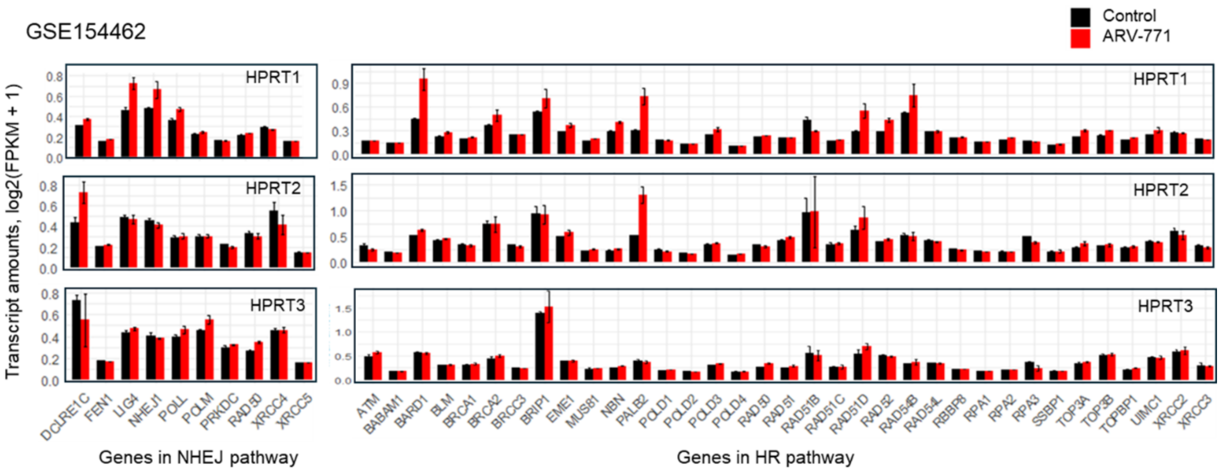

(B)

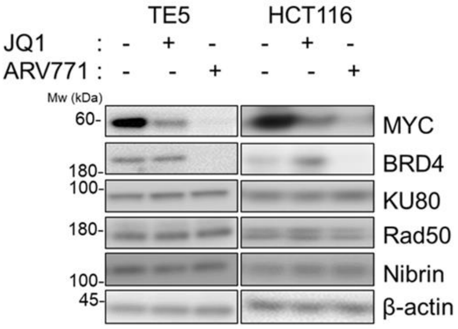

Supplemental Fig. 2. The effects of ARV-771 on the expression levels of genes involved in DNA repair pathway. (A) RNA-seq data from B-cell lymphoma cells (HPRT1, HPRT2, and HPRT3) treated with DMSO or ARV-771 was retrieved from GEO (GSE154462). (B) Western blot analysis of TE5 and HCT116 cells following JQ1 (100 nM, 8 h) and ARV771 (1 μM, 8 h) treatment. Although the downregulation of MYC is prominent in both treatments, the protein levels of KU80, Rad50, and Nibrin as components of DNA damage response remain unchanged.

Supplemental Figure S3

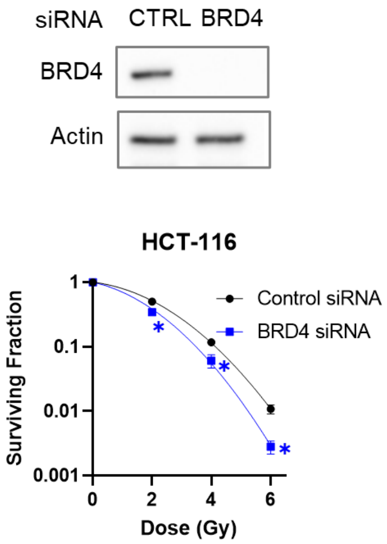

Supplemental Fig. 3. BRD4-silencing works as radiation sensitizer

Western blotting analysis of BRD4 upon siRNA mediated knock-down in HCT116 cell line (Upper). Survival fraction of HCT116 cell lines upon IR alone (Black) and IR combined with BRD4 silencing (Lower).
